# Predicting treatment outcome in depression: an introduction into current concepts and challenges

**DOI:** 10.1007/s00406-022-01418-4

**Published:** 2022-05-19

**Authors:** Nicolas Rost, Elisabeth B. Binder, Tanja M. Brückl

**Affiliations:** 1 Department of Translational Research in Psychiatry, Max Planck Institute of Psychiatry, Kraepelinstraße 2-10, 80804 Munich, Germany; 2 International Max Planck Research School for Translational Psychiatry, Munich, Germany

**Keywords:** Major depressive disorder, Treatment outcome, Predictive modeling, Clinical decision support system, Precision psychiatry

## Abstract

Improving response and remission rates in major depressive disorder (MDD) remains an important challenge. Matching patients to the treatment they will most likely respond to should be the ultimate goal. Even though numerous studies have investigated patient-specific indicators of treatment efficacy, no (bio)markers or empirical tests for use in clinical practice have resulted as of now. Therefore, clinical decisions regarding the treatment of MDD still have to be made on the basis of questionnaire- or interview-based assessments and general guidelines without the support of a (laboratory) test. We conducted a narrative review of current approaches to characterize and predict outcome to pharmacological treatments in MDD. We particularly focused on findings from newer computational studies using machine learning and on the resulting implementation into clinical decision support systems. The main issues seem to rest upon the unavailability of robust predictive variables and the lacking application of empirical findings and predictive models in clinical practice. We outline several challenges that need to be tackled on different stages of the translational process, from current concepts and definitions to generalizable prediction models and their successful implementation into digital support systems. By bridging the addressed gaps in translational psychiatric research, advances in data quantity and new technologies may enable the next steps toward precision psychiatry.

## Introduction

With over 300 million affected people worldwide, depressive disorders have become one of the main causes of disability [1, 2]. Even though there has been an increasing number of studies investigating the optimization of treatment for major depressive disorder (MDD), response rates in patients remain unsatisfactory [3, 4]. In fact, rates have not much improved since the Sequenced Treatment Alternatives to Relieve Depression (STAR*D) study reported in 2006 that only 30% of patients reach the clinical goal of remission, i.e., the absence of symptoms, after the first trial of medication [5]. These numbers need to be taken seriously given the high level of suffering during depressive episodes, the high risk for suicide and comorbidities, and the huge social and economic impact [6, 7]. The question of *what* constitutes the best treatment option for a *specific* patient with a depressive episode under certain individual circumstances is still difficult to answer. Approaches that allow the matching of patients with personalized treatments, often termed ‘precision medicine’, are widely called for in psychiatry [8, 9]. Particularly in early stages of MDD treatment, it is often unclear whether an individual patient will profit most from pharmacotherapy or if other approaches, such as psychotherapy, brain stimulation, or a combination of treatments, might be more beneficial [10]. Models predicting treatment outcome on the basis of individual baseline characteristics can inform the stratification of patients according to their response chances and consequently, the physician’s choice of individualized treatment strategies. In oncology, for example, molecular approaches for tumor characterization have led to the discovery of important subtypes and greatly improved individualized treatments [11, 12]. However, in psychiatry, prediction models have not yielded any reliable and valid (bio)markers that are ready for incorporation into clinical tools to support diagnoses or guide treatment decisions (for a review, see [13]). For the treatment of specific psychiatric disorders, such as MDD, mental health professionals can refer to evidence-based, mostly country-specific, guidelines that have been formulated by a committee of experts, such as the American Psychiatric Association [14] or corresponding organization in other countries (e.g., Germany; [15]). These guidelines typically recommend, depending on depression severity, different initial treatment trials as well as a stepwise increase in treatment intensity if initial treatments fail. To some extent, they also take individual patient characteristics into account by adapting treatment recommendations to specific comorbidity or symptom patterns and the patient’s prior subjective experience with tolerability and efficacy of certain antidepressants. Standardized approaches in the treatment of MDD, such as guideline- and measurement-based [16] treatments, can help to improve treatment success rates [17]. However, treatment guidelines for MDD are also limited by the non-availability of accurate and validated makers of treatment outcome that are needed for the personalization of treatment. Therefore, treatment administration in MDD is often based on the physician’s individual experiences and the patient’s personal preferences [18], potentially adding to the low success rates of MDD treatment [19]. With the current lack of personalized treatment, it is more likely that a chosen treatment will be inefficient than efficient for a certain patient [20].

Thus, a better understanding of individual factors contributing to treatment outcome in MDD continues to be a major topic in psychiatry. The present review summarizes definitions of and issues with the current concepts of treatment outcome and provides an introduction into approaches to study and predict antidepressant outcome in MDD. It focuses on clinical implications from these approaches and on implementations into clinical decision support systems.

## How is treatment outcome in MDD defined?

In the absence of measurable biological indicators of depression severity, it is important to understand how treatment outcome in MDD is commonly defined and how patients are evaluated based on their rate of recovery.

### Changes in symptom severity

In clinical studies, the efficacy of any kind of treatment in MDD as in other psychiatric disorders is routinely assessed with symptom questionnaires, including both clinician-based ratings as well as patient self-ratings. Table 1 summarizes the most typical definitions of treatment outcome based on these ratings. Among the most commonly used scales are the Hamilton Rating Scale for Depression (HDRS; [21]), the Montgomery–Åsberg Depression Rating Scale (MADRS; [22]), the Quick Inventory of Depressive Symptomatology (QIDS; [23]), and the Beck Depression Inventory (BDI; [24]). While the HDRS and MADRS are both clinician-based ratings and require a certain amount of clinical training from the rater [25, 26], the QIDS and BDI are scales based on self-assessments. Even though all these scales were initially created to measure the same construct, i.e., MDD symptom severity, studies have shown that they are not entirely congruent but should rather be used as complementary measures, irrespective of their assessment method [27, 28].

**Table 1:** Definitions of treatment outcome in MDD

Concept	Operationalization in studies	Evaluation
*Symptom severity*	Raw (sum) score derived from a depression severity questionnaire	Continuous measurement Independent from baseline severity As a stand-alone measure (without reference to baseline measure) no clear clinical interpretation
*Change in symptom severity*	Percentage change or difference of (sum) scores derived from depression severity questionnaire between two time points	Continuous measurement Highly dependent on baseline value
*Partial response* Small amount of symptom reduction	Reduction of (sum) score on a depression severity questionnaire usually by 25–49% between treatment start and a specific treatment week	Dichotomous outcome measure (yes/no) Dichotomization by arbitrary threshold Early indicator for stable response later Highly dependent on baseline value
*Response* Considerable amount of symptom reduction	Reduction of (sum) score on a depression severity questionnaire usually by at least 50% between treatment start and a specific treatment week	Dichotomous outcome measure (yes/no) Dichotomization by arbitrary threshold Highly dependent on baseline value Does not necessarily imply that core symptoms have improved
*Remission* Absence of clinically significant amount of symptoms	Depression severity score below a certain threshold. Cut-off values vary by scale: HDRS-17: ≤ 8 / ≤ 7 (e.g., [5, 15]) HDRS-21: ≤ 8 [118] MADRS: ≤ 6 [15] QIDS-SR: ≤ 5 [5] BDI-II: ≤ 9 [5]	Dichotomous outcome measure (yes/no) Dichotomization by arbitrary threshold Reflects the treatment goal Compares an almost symptom-free group to the remainder group
*Symptom trajectories* Pattern of symptom severity changes over time	Identification of patient subgroups with distinct patterns of change in symptom severity over duration of treatment; patients with similar pattern are assigned to the same group; methods often based on unsupervised machine learning	Categorical outcome measure Data-driven method of outcome definition No dichotomization or cut-off value needed Dependent on selected variables and scales Heterogeneous methods and algorithms for subgroup identification
*Treatment resistance* Persistent lack of considerable symptom reduction	Often defined as no significant symptom reduction after at least two adequate antidepressant trials coming from different pharmacological classes; staging approaches	Heterogeneous definitions and staging models (categorical vs. dimensional approaches) Potentially stigmatizing terminology
*Functional recovery* Recovery in daily functioning beyond symptom reduction	Changes in quality of life and disability measures	Important additional measures that imply a broader understanding of recovery (beyond symptom reduction) Often not assessed in clinical studies Subscales partly not applicable in inpatient settings

*HDRS* Hamilton Rating Scale for Depression, *MADRS* Montgomery–Åsberg Depression Rating Scale, *QIDS-SR* Quick Inventory of Depressive Symptomatology (Self-Report), *BDI-II* Beck Depression Inventory-II

Symptom questionnaires are commonly analyzed by adding up their single items into a sum score. Treatment outcome can then be evaluated by simply interpreting this sum score after a certain length of treatment or by comparing it to a baseline score. However, even though the scales are semiquantitative, binary outcome definitions are widely used, the most common ones being ‘response’ and ‘remission’. Treatment response implies a reduction of symptom severity compared to baseline severity by a certain amount (usually by at least 50%), whereas remission requires symptom scores to drop below a certain threshold (e.g., ≤ 7 on the 17-item HDRS; [29]). Since the concept of response relies on the percentage change in symptom severity, it strongly depends on the baseline score. Remission, on the contrary, does not rely on baseline symptom severity at all. From a clinical perspective, remission is the more desired outcome as remitted patients are generally considered symptom-free and, for the time being, fully recovered. Compared to patients who report residual symptoms after treatment (e.g., response without remission), remitters show a reduced risk of subsequent relapse [30, 31].

If depressive symptoms are continuously measured over time, outcome definitions are not restricted to absolute or relative measures, such as response or remission. Instead, trajectories of symptom development over time can be considered to evaluate treatment success. Many longitudinal studies and clinical trials collect data by applying symptom scales on a weekly basis, which allows outcome definitions built on data from more than one or two timepoints. With this information, more refined interpretations of treatment effects can be made for individual patients. Furthermore, symptom trajectories can be used to identify subgroups of patients with similar outcome patterns but different dynamics in change. With increases in computing power, advances in statistical methods and sufficient sample sizes, such approaches are becoming more and more prevalent [32–36].

### Treatment resistance

In contrast to response and remission, non-response and non-remission can be precursors of so-called ‘treatment-resistant depression’ (TRD). Definitions of TRD also depend primarily on scores from symptom questionnaires and are mainly focusing on pharmacotherapy. Even though there is no unique definition [37], TRD is most commonly described as a major depressive episode with no response after two or more trials of adequate antidepressant medication coming from different pharmacological classes [38–40]. Still, although this definition seems to be the most prevalent and a useful common ground, many different definitions exist. Some of them vary fundamentally in their criteria, making them difficult to compare [38, 41].

### Recovery of cognition and daily functioning

Apart from reduction of symptom severity and failed treatment trials, the desired outcome after a depressive episode also includes other aspects of the patient’s recovery. Ideally, patients return to the same (or even a higher) level of well-being as well as to their way of living from before the disorder, including their daily functioning, i.e., their work, social contacts, and general quality of life [42, 43]. This overarching goal of MDD treatment, helping patients to achieve all aspects of recovery, seems to be a stepwise process. For patients with acute moderate or severe episodes, a reduction of symptoms is naturally the first target. Hence, in clinical studies, especially in inpatient settings, symptom severity is more commonly measured than levels of functioning and positive affect [44], the assessments of which are not necessarily well-suited for routine use [19].

Nevertheless, restoration of daily functioning and positive affect are important factors of a holistic picture of recovery. Any potentially impaired cognitive abilities, such as attention, learning, memory, and executive functions [45], should improve, as should components of positive affect, such as optimism and self-confidence [46]. Whereas cognition is routinely assessed using different neurocognitive tests or batteries [47], functional aspects are less well defined [19]. Still, numerous scales and questionnaires with varying foci exist, including the Global Assessment of Functioning [48], the Quality of Life Enjoyment and Satisfaction Questionnaire [49], and the World Health Organization Disability Assessment Schedule [50].

## Prediction models of treatment outcome in MDD

The endeavor of finding indicators of treatment efficacy in MDD has led to a remarkable amount of publications from different psychiatric subfields. A large subset of these have looked at associations of preselected psychological and biological factors with treatment outcome. The main aim hereby was the identification of new (bio)markers using classical statistical approaches, such as regression models with null hypothesis significance testing based on p-values of the investigated predictors. The results from these association studies have been summarized in several systematic reviews and meta-analyses, often focusing on selected data modalities (but see [51, 52]), such as sociodemographic and clinical measures [53], cognitive functioning [54], or blood biomarkers [55]. Table 2 provides a list of these publications grouped by data modality and by their ease of access in clinical practice. Overall, the most consistently identified and most predictive factors were derived from sociodemographic and clinical characteristics [19]. Information on a patient’s social support, their baseline symptom severity, psychiatric comorbidities (e.g., anxiety disorders), or chronicity of the disorder, for instance, have repeatedly been associated with MDD treatment outcome [51–53]. However, an important shortcoming of these results is that none of the identified measures has been proven informative enough to sufficiently predict treatment outcome on their own.

**Table 2:** Different measurement techniques used in psychiatric research and corresponding examples of derived factors associated with antidepressant treatment effects

Measurement technique	Requirements	Example indicators of antidepressant treatment outcome
Easily accessible and usable		
Questionnaires and clinician-based ratings or interviews	Manuals (Clinical training)	Social demographics [53, 119, 120] Symptom profiles [53, 119, 120] Comorbidities [121, 122] Personality traits [123] Exposure to environmental risk factors, e.g., childhood abuse [124–126]
Tests and tasks	Manuals Technical devices for digital implementations	Cognitive functioning [54] Emotional processing [127, 128]
Technically feasible but additional efforts and expenses needed		
Blood draw or saliva sampling for established parameters	Medical training and equipment Laboratory capacities	Immune parameters, e.g., cytokines [129–131] Metabolites [132] Pharmacogenomic testing [133–135]
Dynamic function tests	Medical training and equipment Laboratory capacities	HPA-axis regulation [136, 137]
Technically feasible but high complexity and expenses		
Genotyping pipelines (based on blood draw or saliva sampling or other biospecimen)	Medical training and equipment Laboratory capacities Computational expertise and resources	Candidate genes without established testing [138, 139] Genome-wide associations [140–145] Polygenic risk scores [146] Epigenetic, transcriptomic and metabolomic markers [147–151]
Technical recording devices	Special equipment Technical training and expertise	Neuroimaging [152–155] Electroencephalographic markers [156, 157] Peripheral physiological markers [158]

Measurements are grouped by their accessibility and usability for routine clinical practice and licensed physicians. Note that this table is neither exhaustive nor based on a systematic literature search but meant to show exemplary indicators and their translational value

This issue has led to a “new generation” of studies which aim at creating prediction models based on a multitude of variables. These models use machine learning (ML) methods, mainly supervised learning with classification algorithms such as regularized logistic regression or tree-based methods [56], to combine the effects of many variables and to increase predictive accuracy. Hence, they do not necessarily focus on the identification of new predictors of treatment outcome but rather try to find the best combination of variables to maximize their predictive power. A clear and comprehensive review on ML models and their value for predicting treatment outcome in psychiatry was recently published [57], as well as a systematic review and meta-analysis of these approaches in MDD specifically [58]. Crucially, the development of such models needs to include some kind of validation in order assure that predictions are not specific to the data they were created from but also generalize to new data. Validation is often performed by dividing the initial data set into subsamples (e.g., training sample and validation sample) or by testing the model’s performance on a completely independent sample [59]. Furthermore, sufficiently large data sets in terms of sample size are required to guarantee robustness and generalizability of the predictions. The majority of predictive ML models of MDD treatment outcome have thus been created on data from large patient cohorts coming either from clinical trials (such as STAR*D [60, 61], Genome-based Therapeutic Drugs for Depression [62, 63], or Establishing Moderators and Biosignatures of Antidepressant Response for Clinical Care in Depression [64]), or from observational studies (such as the Munich Antidepressant Response Signature project [32] or the Netherlands Study of Depression and Anxiety (NESDA) [65, 66]). Since clinical trials usually compare different treatment arms (or treatment against placebo), the resulting predictions are likely be treatment-specific and may not be readily applied to other treatments [60, 62]. Observational studies, on the other hand, follow a more naturalistic approach by observing patients who are treated based on routine clinical decisions, which might lead to more heterogeneity in the data [67, 68]. In general, prediction models of MDD treatment outcome based on sample sizes of at least several hundred patients (e.g., [60–63]) can predict treatment outcome (most often response vs. non-response or remission vs. non-remission) with moderate to good accuracies of 65%–75% [58]. This means that up to three quarters of ‘true’ responders/remitters are recognized as such by these prediction models. Most models that have been published so far have confirmed that the most reliable predictors of MDD treatment outcome come from established clinical and sociodemographic factors that had already been identified in earlier studies, such as initial symptom severity (e.g., [32, 36, 60, 62]), number and duration of depressive episodes (e.g., [32, 60]), personality traits (e.g., [32, 66]), as well as employment status and education (e.g., [61, 66]). However, only few studies exist that have assessed the additional value of other data modalities by comparing the performance of a multimodal model to a model using sociodemographic or clinical variables only. We here provide two examples of studies that have followed this approach using large sample sizes (at least several hundred samples) and ML methods. Iniesta et al. [63] showed that a prediction model combining demographic and clinical variables (e.g., depressive symptom scores, medication status, and stressful life events) with over 500,000 genetic markers (single nucleotide polymorphisms and copy number variants) led to slightly more accurate predictions (area under the receiver operating characteristic curve (AUC) of 0.77) than a model trained on the non-genetic variables only (AUC of 0.74; [62]). Similarly, Dinga et al. [66] compared a prediction model combining clinical and biological data (primarily somatic health measures, inflammatory and metabolic markers) to models including only one of the available predictor domains. Across all comparisons, the full model containing all variables performed better than the alternative models. The largest differences occurred when the alternative model was based on biological measures only, the smallest differences when it was based on depressive symptom severity scores (differences in AUC of 0.01–0.05). These results suggest that even though adding biological markers to prediction models can lead to increases in performance, their additional value on top of clinical data still remains small.

## Clinical decision support systems in psychiatry

A suitable instrument to transfer predictive models from research into clinical practice is a Clinical Decision Support System (CDSS). CDSSs are any kinds of computer systems that work with clinical data or knowledge and are set up to assist healthcare professionals in decision processes [69]. These decisions can refer to both diagnosing a patient and selecting the best treatment [70]. Concretely, a patient’s characteristics enter a CDSS to be evaluated based on implemented clinical knowledge in order to return recommendations to the clinicians [71]. Hence, these systems can improve clinical processes and help healthcare professionals benefit from scientific findings [72].

CDSSs have been used successfully in many medical disciplines (for a review, see [73]), but use in psychiatry or mental health is lagging behind. However, some systems have been developed for the diagnoses of mental disorders, e.g., for attention deficit hyperactivity disorder [74], MDD and anxiety disorders [75], subtypes of schizophrenia [76], or a broader range of disorders [77]. Other systems were designed more specifically and can also be of value for MDD, such as the NetDSS [78], a web-based CDSS with various functions, from patient registry to clinical outcome monitoring. An elegant tool for physicians and patients was set up by Henshall et al. [79]. They developed a recommendation system and tested it on a focus group comprising physicians, caregivers, and patients with several mental disorders, including MDD. By entering basic sociodemographic and clinical variables as well as by setting preferences for potential side effects, the software returned a graphical illustration of recommended interventions and their corresponding probabilities of effectiveness. A benefit of such a tool is that it uses individual data to tailor a treatment to each patient. Similarly, a few commercial tools have been developed lately, promoting improvements of treatment efficacy for mental disorders using individual patient data and predictive models [80–82].

Ultimately, such predictive systems can enhance personalized treatment, e.g., by indicating from the beginning which medication has the highest probability to lead to a beneficial response. Moreover, these tools can save physicians time and increase preciseness of clinical judgements [83, 84].

## Current challenges and unmet needs

**Fig. 1:**
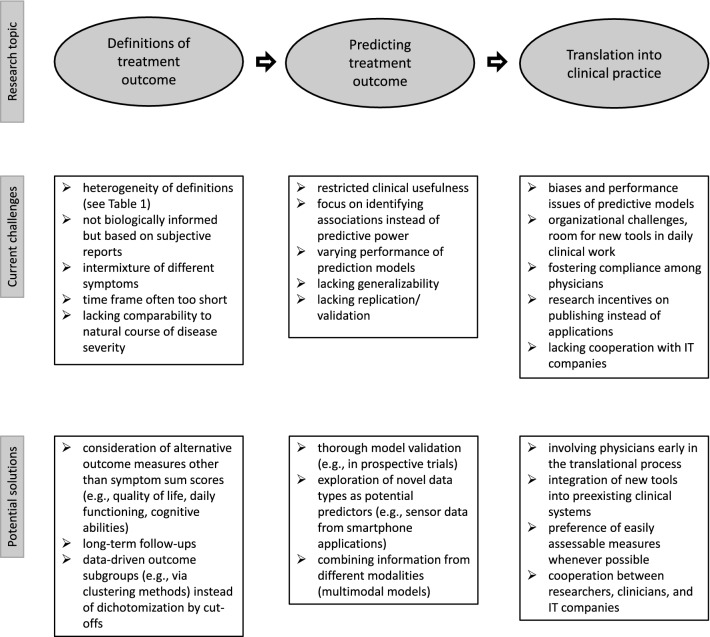
Current challenges with respect to different stages of research on treatment outcome in MDD patients and its translation into clinical practice

With the increasing interest in precision psychiatry and outcome prognosis, many efforts have been invested in this field of research. Nonetheless, the core problem in translational psychiatry remains: translations of research findings into daily clinical work, in such a way that patients and clinicians could directly benefit from them, are practically non-existent. Due to the lack of validated tests as guidance for personalized medication, treatment administration still has to rely on generic guidelines and physicians’ personal judgements. The potential solution appears to be twofold: first, robust (bio)markers of treatment efficacy need to be identified and built into prognostic models. Subsequently, if models are proven useful, the second step will be their translation into new tools for clinicians. The main issues and current challenges in this translational process as well as potential solution approaches are outlined below. Additionally, they are illustrated in Fig. 1.

### Challenges in concepts and definitions

Up to 16,400 potential symptom combinations can lead to a diagnosis of MDD [85], which might essentially be a conglomerate of many different pathophysiologies [86]. Moreover, MDD shows a high degree of comorbidity with other mental disorders, both cross-sectionally [87–89] and over time [90]. Longitudinal studies, especially using registry data [91], have shown large variability of diagnoses across lifetime which is why a cross-sectional focus on MDD diagnosis might miss relevant longitudinal information that discriminates among disorder subtypes. Hence, transdiagnostic and longitudinal approaches (e.g., assessing lifetime disorders in diagnostic interviews) should be considered in clinical studies.

A second challenge is posed by the measurements and definitions of antidepressant outcome (see Table 1). Unlike other medical disciplines, which provide objective biological measures of disease severity or treatment success, psychiatry defines clinical outcomes on subjective ratings (self-reported or clinician-rated). However, some of the most common ratings were shown to lack reliability [27, 92, 93] and to be incongruent among themselves, meaning that they do not measure exactly the same construct and are thus not fully comparable [28]. These issues limit the validity of findings and the generalizability from one outcome scale to others. Moreover, ratings of depressive symptom severity, such as the HDRS, the QIDS, or the BDI, evaluate many different symptoms and aspects of MDD, all influencing the respective sum score. It is possible for patients to show a 50% reduction of the sum score and be classified as responders, even when none of the core symptoms of MDD (depressed mood or reduced interest/pleasure in activities) have improved. Furthermore, patients with the same overall severity score can show very different symptom profiles, and have thus very different subjective experiences of their disorder. This important information gets lost when sum score data are used [94]. Explicitly differentiating between symptoms instead of using sum scores could help to identify indicators of specific symptoms and could thus lead toward more targeted treatments [95].

Moreover, antidepressant outcome is often defined as (partial) response or remission (see Table 1). Both terms represent artificially dichotomized variables, created based on more or less arbitrary cut-off values on a continuous scale, that is, the respective sum score (for remission) or the difference in sum scores (for response) on a symptom scale. Dichotomizing continuous variables always brings certain risks and comes with loss of information [96]. Consider two patients with very similar symptom scores during the course of treatment, e.g., symptom reductions of 55% and 45%, respectively. According to the common definition of treatment response, the first patient would be classified as a ‘responder’ whereas the second patient would be treated as a ‘non-responder’. In fact, the second patient would be categorized together with patients who do not show any symptom reduction at all. Classifying patients in a data-driven manner, e.g., using clustering techniques to create more homogenous outcome classes, might be a promising alternative that has already been implemented in several studies [32–35]. Still, the resulting outcome groups strongly depended on the selected variables and the chosen clustering method. Hence, the number of identified groups varied, e.g., from five [33] to seven [32, 34] up to nine [35]. These discrepancies challenge their clinical usefulness as the obtained classes are likely not generalizable to most other settings. Nevertheless, especially if more than one type of outcome measure is available, clustering methods might be a good way to combine information and identify subgroups.

Another issue with common measurements of treatment efficacy is the time frame. Patients in clinical trials are often measured over a few weeks only. Especially in disorders such as MDD, which can appear recurrently and show a risk of chronification [97], it is important to follow up on patients after a longer period of time. This could help differentiate between temporary improvements and long-term recovery. In the NESDA sample, 22% of initially remitted patients developed a recurrent episode within the following 2 years [98]. Identifying these at-risk patients early on might help to prevent subsequent episodes by scheduling regular checkups and implementing prevention strategies [99].

Even in the absence of reliable (biological) alternatives, sum scores on symptom questionnaires alone do not seem to be the most specific and clinically meaningful measures [95, 100]. In a recent online survey, MDD patients, informal caregivers, and healthcare professionals were asked to indicate outcome domains that matter most in their opinion. They identified not only depressive symptoms but also domains of functioning, healthcare organization, and social representation, many of which are not measured in most clinical studies, let alone included in depression rating scales [44, 101], highlighting the importance of including patient centered outcomes. Another research team explicitly differentiated between opinions from doctors and patients [102]. Their survey revealed that physicians mainly considered alleviation of depressive symptoms to be most important for relief and cure from MDD whereas patients rather focused on rehabilitation of positive affect. These results suggest that definitions and measures of treatment outcome should go beyond plain ratings of symptom changes and need to be broadened and potentially lengthened [42]. Relevant assessment instruments for many different domains of MDD characterization, including neurocognition, functioning and quality of life, as well as their suitability for routine clinical use have recently been reviewed [19] and should be considered when measuring treatment outcome in future studies.

Finally, novel objective measures that do not rely on subjective self- or external reports, such as behavioral and functional data generated by smartphones, wearables or other digital devices, could be of further value [103]. As long as no direct biological measure of treatment outcome exists, personal data collected from mobile devices, i.e., ‘digital phenotyping’, might become a promising alternative [104]. Ecological momentary assessments, actimetry, speech characteristics, or movement patterns, for instance, can be continuously and mainly passively collected in large amounts and in high temporal resolution. Sensor data and other information from wearable devices like smartphones have already been successfully applied in psychiatric research, especially in combination with ML and deep learning [103]. Future studies will need to prove if they can contribute to a deeper and broader characterization of treatment outcome and MDD.

### Challenges for prediction models

Except for a few psychometric and sociodemographic factors, there are still no robust or well replicated predictors of treatment outcome. Apart from a few promising pharmacogenetic tests [81, 105], no biological measures qualify as stable biomarkers nor are they used in clinical practice. Associations between specific measurements and treatment outcome are often of limited prognostic value as statistically significant associations do not guarantee accurate and robust predictions. Therefore, the focus has started to shift from testing associations to improving predictions in order to forecast what is most beneficial for an individual patient and to personalize clinical decision-making [106].

Predictive ML models tackle this issue as they are built to be as accurate and robust as possible. The robustness of a model should be assessed by validating it on an independent data set [57], ideally by testing its performance and safety on new patients in a prospective clinical study. Nonetheless, several prediction models were not validated on external data sets at all (e.g., [32, 64, 65]). Others were less predictive when they were applied to other classes of antidepressants, suggesting that the identified predictors of treatment outcome might be agent-specific [60, 62]. In addition, the main target variables in studies using ML were response and remission in their binary form [58], the downsides of which have already been discussed. Furthermore, psychiatric data often face the problem of high dimensionality while samples sizes remain relatively small [107]. This is often referred to as the ‘curse of dimensionality’: the more variables a data set contains, the more the sample size needs to increase (per variable) to allow reliable results [59]. Otherwise, resulting prediction models are likely to be biased and therefore need to be carefully validated on independent data to ensure their reliability. Moreover, prediction models based on biological data often only show restricted translatability into clinical practice as they require precisely preprocessed data from time-consuming and expensive measurements. A prerequisite for a successful translation of a predictive model into clinical practice is that it consists of parameters that can be routinely accessed by a licensed physician without producing a lot of extra costs. Psychological and clinical features as well as sociodemographic information can be evaluated easily by any trained clinician or via self-ratings. On the other hand, as indicated in Table 2, many biological measures, i.e., potential biomarkers, are comparatively expensive or hard to assess for physicians in common clinical settings. This is especially the case for neuroimaging, omics data, and endocrinological markers derived from a challenge test, for instance. Such parameters should only be preferred over less costly data modalities, e.g., questionnaire data, if their predictive performance is notably higher and thus justifies the additional expenses. Making use of other objective measures, such as data collected from smartphones and other wearable devices, might become a promising alternative [103]. Their collection would be economical and profitable for researchers as well as less time-consuming and free of stress for patients.

In summary, well-performing and externally validated ML models are promising tools for future psychiatric practice [59], including the prognosis of treatment outcome in MDD.

### Challenges for CDSSs

In order to translate predictive models into digital tools for everyday clinical use, CDSSs could be of help. Iniesta et al. [108] sketched a concise outline of the workflow for designing and choosing predictive models and, crucially, explained how to bring them into CDSSs. Still, as appealing as the idea of such publicly used tools might sound, they have not yet become prevalent in healthcare institutions.

The main challenge in MDD outcome prediction seems to be the lack of powerful models and established predictive patient characteristics. As outlined above, predictive models are still not robust and generalizable enough to guide daily clinical decisions. Only if additional value coming from a predictive model is proven, will an implementation into a CDSSs lead to a successful supporting device. Biases in such systems, for instance, were shown to lead to underestimations of their effectiveness [109], high non-compliance rates among users [73], and even to wrong diagnoses by physicians [110]. This is particularly concerning given that working with a CDSS might influence clinicians in their decisions later on even when they are not explicitly using the system anymore [111].

Furthermore, before CDSSs can be fully implemented into clinical workflows, substantial ethical challenges need to be considered. Apart from data protection, which needs to be assured, questions regarding liability and responsibility for treatment decisions have to be addressed, especially when it comes to disagreement between physicians and support systems. Also, human interactions, conversations and relations between patients and mental health professionals play an important role, not only in psychiatric care [112, 113]. Further necessary ethical considerations have been summarized by Chekroud et al. [57].

Due to these problems, a number of factors needed to sustainably establish CDSSs in clinical settings should be considered [73]: First, apart from having appealing visual designs and being user-friendly, the system should implement personalized, transparent, and reliable recommendations as well as comprehensive overviews for each patient. Second, physicians should keep the authority over treatment decisions and should still oversee algorithmic outputs [114]. They should be involved in the development of the system, receive training and not have to make adaptations in their daily working processes in order to use the application. Third, to circumvent organizational obstacles, CDSSs should be integrated into preexisting clinical computerized systems, such as electronic medical records or physician order entries [73].

Ultimately, however, the main incentive in research seems to remain the publication of novel findings, indeed funding for the translation of existing findings into applications and technical devices is often more difficult to obtain [115, 116]. Therefore, interdisciplinary work is needed, bringing together scientists, clinicians and, e.g., information technologists for successful development of CDSSs.

## Conclusion

Tackling the medical treatment of MDD and increasing treatment efficacy have always been major challenges in psychiatric research. In this narrative review, we summarized current approaches to operationalize and predict treatment outcome in MDD. We highlighted findings from ML approaches and discussed their implementation into CDSSs. To date, numerous studies have investigated and discovered associations between biological and phenotypic patient characteristics and treatment outcome, producing growing evidence for potential underlying mechanisms. Large patient cohort data and ML methods have additionally produced predictive models with promising accuracies (e.g., [32, 36, 60, 62, 64, 65]). Nevertheless, psychiatry has made comparatively little progress in applying the acquired knowledge into daily clinical work and in personalizing decisions based on empirically derived patient characteristics.

The main issue of this lacking translation seems to be the absence of robust and generalizable predictors of treatment outcome, especially of biological and other objectively measurable markers. Further quantitative characterizations of patients might help to identify more robust predictors and could provide support in medical decisions, such as choosing the most beneficial treatment for individual patients or subgroups of patients [117]. Once reliable indicators and prognostic models are established, the next challenge will be their implementation into clinical practice. Efficient systems with clear interpretation of results need to be introduced and made available for healthcare professionals. CDSSs can be useful tools to implement tests and predictive models to guarantee benefits for physicians and patients. To make this happen, research funding needs to put more emphasis on translational systems, i.e., the development of target-oriented and clinically useful applications. Cooperation with companies specialized in health information technologies might be of particular use for this endeavor. Finally, there needs to be a shift in psychiatry toward a data-driven stratification of patients as well as more precise, personalized treatments based on individual patient data.
